# 4-Chloro­anilinium perchlorate–18-crown-16 (1/1)

**DOI:** 10.1107/S1600536811048859

**Published:** 2011-11-23

**Authors:** Chun-Hua Yu

**Affiliations:** a Ordered Matter Science Research Center, Collenge of Chemistry and Chemical Engineering, Southeast University, Nanjing 211189, People’s Republic of China

## Abstract

In the title compound, C_6_H_7_ClN^+^·ClO_4_^−^·C_12_H_24_O_6_, the cation forms a 1:1 complex with the crown ether, *viz* [C_6_H_7_ClN-(18-crown-6)]^+^, in which the –NH_3_^+^ unit nests in the crown and inter­acts with it through bifurcated N—H⋯O hydrogen bonding. All constituents of the structure have crystallographically imposed mirror symmetry except for the H atoms of the –NH_3_^+^ group which are disordered across the mirror.

## Related literature

The title compound was synthesized as part of a study aimed at finding new ferroelectric materials. For general background to ferroelectric compounds, see: Fu *et al.* (2009 ▶); Ye *et al.* (2006 ▶); Zhang, Xiong *et al.* (2008 ▶); Zhang, Ye *et al.* (2010 ▶). For background to crown ether/ammonium ion complexes, see: Fender *et al.* (2002 ▶); Kryatova *et al.* (2004 ▶).
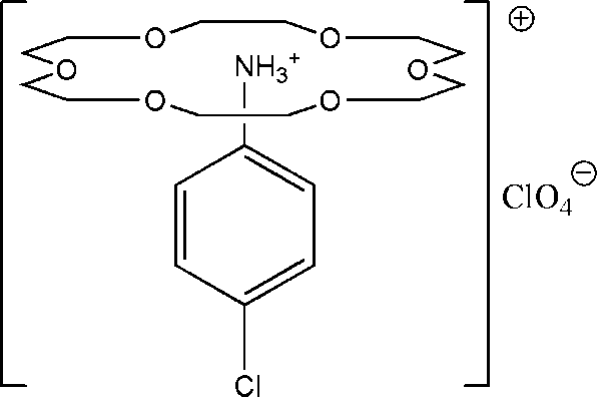


## Experimental

### 

#### Crystal data

C_6_H_7_ClN^+^·ClO_4_^−^·C_12_H_24_O_6_
*M* *_r_* = 492.34
Orthorhombic, 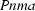

*a* = 15.726 (3) Å
*b* = 11.525 (2) Å
*c* = 12.896 (3) Å
*V* = 2337.3 (8) Å^3^
*Z* = 4
Mo *K*α radiation
μ = 0.33 mm^−1^
*T* = 293 K
0.35 × 0.32 × 0.28 mm

#### Data collection

Rigaku SCXmini diffractometer
Absorption correction: multi-scan (*CrystalClear*; Rigaku, 2005 ▶) *T*_min_ = 0.891, *T*_max_ = 0.912
23203 measured reflections
2820 independent reflections
2231 reflections with *I* > 2σ(*I*)
*R*_int_ = 0.048

#### Refinement

*R*[*F* ^2^ > 2σ(*F* ^2^)] = 0.045
*wR*(*F* ^2^) = 0.114
*S* = 1.08
2820 reflections
155 parameters
H-atom parameters constrained
Δρ_max_ = 0.25 e Å^−3^
Δρ_min_ = −0.36 e Å^−3^

### 

Data collection: *CrystalClear* (Rigaku, 2005 ▶); cell refinement: *CrystalClear*; data reduction: *CrystalClear*; program(s) used to solve structure: *SHELXS97* (Sheldrick, 2008 ▶); program(s) used to refine structure: *SHELXL97* (Sheldrick, 2008 ▶); molecular graphics: *SHELXTL* (Sheldrick, 2008 ▶); software used to prepare material for publication: *SHELXL97*.

## Supplementary Material

Crystal structure: contains datablock(s) I, global. DOI: 10.1107/S1600536811048859/mw2036sup1.cif

Structure factors: contains datablock(s) I. DOI: 10.1107/S1600536811048859/mw2036Isup2.hkl

Additional supplementary materials:  crystallographic information; 3D view; checkCIF report

## Figures and Tables

**Table 1:** Hydrogen-bond geometry (Å, °)

*D*—H⋯*A*	*D*—H	H⋯*A*	*D*⋯*A*	*D*—H⋯*A*
N1—H1*A*⋯O4	0.89	2.14	2.896 (3)	142
N1—H1*A*⋯O3^i^	0.89	2.21	2.9311 (17)	138
N1—H1*B*⋯O2^i^	0.89	2.14	2.8952 (18)	143
N1—H1*B*⋯O1	0.89	2.18	2.870 (3)	134
N1—H1*C*⋯O2	0.89	2.15	2.8952 (18)	140
N1—H1*C*⋯O3	0.89	2.20	2.9311 (17)	139

Symmetry code: (i) 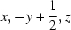.

**Fig. 1.**
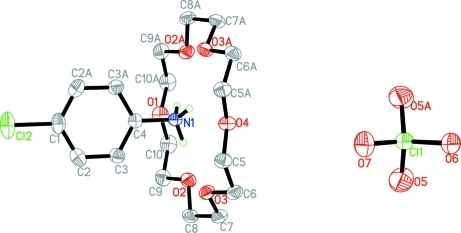
Perspective view of (I), showing the atom-numbering scheme. Displacement ellipsoids are drawn at the 30% probability level.

**Fig. 2.**
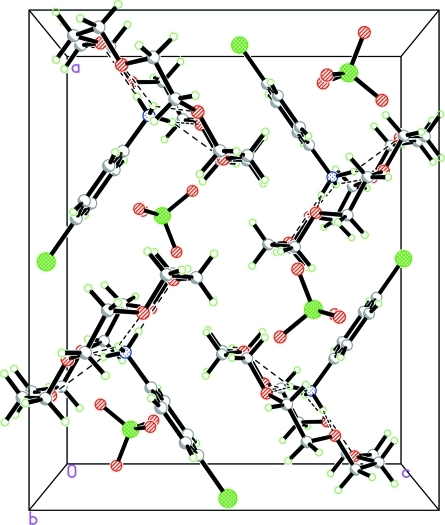
A view of the packing of the title compound down the b axis. Dashed lines indicate hydrogen bonds.

## supplementary crystallographic information

### Comment

We synthesized the title compound, (I), with the aim of finding new ferroelectric materials (Fu *et al.*, 2009; Ye *et al.*, 2006; Zhang, Xiong *et al.*, 2008; Zhang, Ye *et al.*, 2010). There is currently a significant interest in crown ethers because of their ability to form noncovalent, hydrogen bonding complexes with ammonium cations both in the solid state and in solution (Fender *et al.*, 2002; Kryatova *et al.*, 2004). In the crystal, the *p*-chloroanilium cations and 18-crown-6 molecules are associated *via* hydrogen bonding with the –NH_3_^+^ group forming bifurcated hydrogen bonds with all six O atoms of the crown ether molecule (Figure 1, Table 1). Despite the disorder in the –NH_3_^+^ group, it is clear that in each orientation the cation forms three bifurcated hydrogen bonds.

### Experimental

*p*-chloroaniline (1.28 g, 10 mmol) was dissolved in *N*,*N*-dimethyl formamide(DMF) (10 ml) to which an aqueous solution of perchloric acid was dropped slowly with stirring until the pH of the solution was *ca* 7. 18-crown-6 (2.64 g 10 mmol) was added to the solution and more DMF was added until the initial precipitate dissolved. The solution was filtered to a clean beaker and massive crystals of (I) were obtained *via* slow evaporation of the DMF solution at room temperature over several weeks.

### Refinement

H atoms attached to C were placed in calculated positions (C—H = 0.93 Å for C*sp^2^* atoms and 0.97 Å for C*sp^3^* atoms) while those attached to N were placed in positions derived from a difference map and the N—H distances adjusted to 0.89 Å. All were included as riding contributions with *U*_iso_ values tied to those of the attached atoms (*U*_iso_ = 1.2*U*eq(C*sp^2^*/N) and 1.5*U*eq(C*sp^3^*)).

### Crystal data

**Table:**

C_6_H_7_ClN^+^·ClO_4_^−^·C_12_H_24_O_6_	*F*(000) = 1040
*M**_r_* = 492.34	*D*_x_ = 1.399 Mg m^−^^3^
Orthorhombic, *P**n**m**a*	Mo *K*α radiation, λ = 0.71073 Å
Hall symbol: -P 2ac 2n	Cell parameters from 2820 reflections
*a* = 15.726 (3) Å	θ = 3.0–27.5°
*b* = 11.525 (2) Å	µ = 0.33 mm^−^^1^
*c* = 12.896 (3) Å	*T* = 293 K
*V* = 2337.3 (8) Å^3^	Block, colorless
*Z* = 4	0.35 × 0.32 × 0.28 mm

### Data collection

**Table:**

Rigaku SCXmini diffractometer	2820 independent reflections
Radiation source: fine-focus sealed tube	2231 reflections with *I* > 2σ(*I*)
graphite	*R*_int_ = 0.048
ω scans	θ_max_ = 27.5°, θ_min_ = 3.0°
Absorption correction: multi-scan (*CrystalClear*; Rigaku, 2005)	*h* = −20→20
*T*_min_ = 0.891, *T*_max_ = 0.912	*k* = −14→14
23203 measured reflections	*l* = −16→16

### Refinement

**Table:**

Refinement on *F*^2^	Secondary atom site location: difference Fourier map
Least-squares matrix: full	Hydrogen site location: inferred from neighbouring sites
*R*[*F*^2^ > 2σ(*F*^2^)] = 0.045	H-atom parameters constrained
*wR*(*F*^2^) = 0.114	*w* = 1/[σ^2^(*F*_o_^2^) + (0.0418*P*)^2^ + 0.9195*P*] where *P* = (*F*_o_^2^ + 2*F*_c_^2^)/3
*S* = 1.08	(Δ/σ)_max_ = 0.001
2820 reflections	Δρ_max_ = 0.25 e Å^−^^3^
155 parameters	Δρ_min_ = −0.36 e Å^−^^3^
0 restraints	Extinction correction: *SHELXL97* (Sheldrick, 2008), Fc^*^=kFc[1+0.001xFc^2^λ^3^/sin(2θ)]^-1/4^
Primary atom site location: structure-invariant direct methods	Extinction coefficient: 0.0159 (11)

### Special details

**Table:**

Geometry. All e.s.d.'s (except the e.s.d. in the dihedral angle between two l.s. planes) are estimated using the full covariance matrix. The cell e.s.d.'s are taken into account individually in the estimation of e.s.d.'s in distances, angles and torsion angles; correlations between e.s.d.'s in cell parameters are only used when they are defined by crystal symmetry. An approximate (isotropic) treatment of cell e.s.d.'s is used for estimating e.s.d.'s involving l.s. planes.
Refinement. Refinement of *F*^2^ against ALL reflections. The weighted *R*-factor *wR* and goodness of fit *S* are based on *F*^2^, conventional *R*-factors *R* are based on *F*, with *F* set to zero for negative *F*^2^. The threshold expression of *F*^2^ > σ(*F*^2^) is used only for calculating *R*-factors(gt) *etc*. and is not relevant to the choice of reflections for refinement. *R*-factors based on *F*^2^ are statistically about twice as large as those based on *F*, and *R*- factors based on ALL data will be even larger.

### Fractional atomic coordinates and isotropic or equivalent isotropic displacement parameters (Å^2^)

**Table:**

	*x*	*y*	*z*	*U*_iso_*/*U*_eq_	Occ. (<1)
C1	0.91237 (18)	0.2500	0.5933 (2)	0.0575 (8)	
C2	0.87729 (13)	0.1459 (2)	0.62262 (16)	0.0587 (5)	
H2	0.9016	0.0763	0.6014	0.070*	
C3	0.80513 (12)	0.14605 (17)	0.68421 (15)	0.0500 (5)	
H3	0.7808	0.0764	0.7052	0.060*	
C4	0.76968 (15)	0.2500	0.71422 (17)	0.0378 (5)	
C5	0.53619 (13)	0.14754 (16)	0.59540 (14)	0.0496 (5)	
H5A	0.5865	0.1403	0.5524	0.059*	
H5B	0.4868	0.1508	0.5504	0.059*	
C6	0.52948 (12)	0.04615 (17)	0.66648 (16)	0.0512 (5)	
H6A	0.4820	0.0570	0.7135	0.061*	
H6B	0.5194	−0.0239	0.6266	0.061*	
C7	0.59921 (13)	−0.04958 (17)	0.80440 (16)	0.0544 (5)	
H7A	0.5829	−0.1238	0.7751	0.065*	
H7B	0.5557	−0.0262	0.8535	0.065*	
C8	0.68241 (13)	−0.06071 (17)	0.85833 (16)	0.0545 (5)	
H8A	0.6801	−0.1240	0.9079	0.065*	
H8B	0.7269	−0.0775	0.8084	0.065*	
C9	0.77936 (13)	0.0436 (2)	0.96481 (17)	0.0600 (6)	
H9A	0.8262	0.0451	0.9158	0.072*	
H9B	0.7837	−0.0269	1.0056	0.072*	
C10	0.78372 (15)	0.1468 (2)	1.03395 (16)	0.0644 (6)	
H10A	0.7344	0.1485	1.0791	0.077*	
H10B	0.8342	0.1423	1.0770	0.077*	
Cl1	0.10405 (4)	0.2500	0.20475 (5)	0.0469 (2)	
Cl2	1.00340 (6)	0.2500	0.51600 (8)	0.0976 (4)	
N1	0.69437 (12)	0.2500	0.78054 (15)	0.0387 (5)	
H1A	0.6518	0.2853	0.7478	0.046*	0.50
H1B	0.7058	0.2875	0.8392	0.046*	0.50
H1C	0.6796	0.1772	0.7949	0.046*	0.50
O1	0.78626 (12)	0.2500	0.97279 (13)	0.0544 (5)	
O2	0.70060 (8)	0.04546 (12)	0.91065 (11)	0.0527 (4)	
O3	0.60630 (8)	0.03499 (11)	0.72394 (10)	0.0478 (3)	
O4	0.54115 (12)	0.2500	0.65597 (13)	0.0455 (4)	
O5	0.11758 (11)	0.14801 (15)	0.26549 (15)	0.0858 (6)	
O6	0.01894 (12)	0.2500	0.16435 (17)	0.0607 (6)	
O7	0.16221 (14)	0.2500	0.11884 (19)	0.0741 (6)	

### Atomic displacement parameters (Å^2^)

**Table:**

	*U*^11^	*U*^22^	*U*^33^	*U*^12^	*U*^13^	*U*^23^
C1	0.0407 (14)	0.090 (2)	0.0422 (15)	0.000	−0.0008 (12)	0.000
C2	0.0585 (12)	0.0672 (14)	0.0505 (11)	0.0166 (10)	0.0025 (10)	−0.0023 (10)
C3	0.0553 (11)	0.0468 (11)	0.0479 (10)	0.0032 (9)	0.0018 (9)	0.0011 (8)
C4	0.0391 (12)	0.0444 (13)	0.0300 (11)	0.000	−0.0071 (10)	0.000
C5	0.0565 (11)	0.0473 (11)	0.0449 (10)	−0.0017 (9)	−0.0074 (9)	−0.0085 (8)
C6	0.0509 (11)	0.0433 (11)	0.0593 (12)	−0.0099 (8)	−0.0062 (9)	−0.0063 (9)
C7	0.0625 (12)	0.0391 (10)	0.0615 (12)	−0.0065 (9)	0.0045 (10)	0.0074 (9)
C8	0.0642 (12)	0.0384 (10)	0.0608 (12)	0.0091 (9)	0.0062 (10)	0.0101 (9)
C9	0.0535 (12)	0.0660 (14)	0.0605 (13)	0.0097 (10)	−0.0093 (10)	0.0190 (11)
C10	0.0638 (13)	0.0852 (17)	0.0443 (11)	0.0034 (12)	−0.0125 (10)	0.0145 (11)
Cl1	0.0432 (3)	0.0385 (3)	0.0590 (4)	0.000	−0.0009 (3)	0.000
Cl2	0.0602 (5)	0.1460 (10)	0.0868 (7)	0.000	0.0281 (5)	0.000
N1	0.0406 (11)	0.0353 (10)	0.0402 (11)	0.000	−0.0047 (9)	0.000
O1	0.0623 (12)	0.0639 (13)	0.0371 (10)	0.000	−0.0077 (9)	0.000
O2	0.0505 (8)	0.0460 (8)	0.0615 (8)	0.0089 (6)	−0.0078 (6)	0.0064 (6)
O3	0.0491 (7)	0.0395 (7)	0.0549 (8)	−0.0047 (5)	−0.0017 (6)	0.0056 (6)
O4	0.0579 (11)	0.0386 (10)	0.0402 (9)	0.000	−0.0079 (8)	0.000
O5	0.0794 (11)	0.0744 (12)	0.1034 (13)	0.0009 (9)	−0.0148 (10)	0.0373 (10)
O6	0.0463 (11)	0.0551 (12)	0.0807 (14)	0.000	−0.0060 (10)	0.000
O7	0.0584 (13)	0.0833 (16)	0.0807 (15)	0.000	0.0156 (12)	0.000

### Geometric parameters (Å, °)

**Table:**

C1—C2	1.374 (3)	C8—O2	1.426 (2)
C1—C2^i^	1.374 (3)	C8—H8A	0.9700
C1—Cl2	1.744 (3)	C8—H8B	0.9700
C2—C3	1.385 (3)	C9—O2	1.422 (2)
C2—H2	0.9300	C9—C10	1.489 (3)
C3—C4	1.377 (2)	C9—H9A	0.9700
C3—H3	0.9300	C9—H9B	0.9700
C4—C3^i^	1.377 (2)	C10—O1	1.427 (2)
C4—N1	1.461 (3)	C10—H10A	0.9700
C5—O4	1.418 (2)	C10—H10B	0.9700
C5—C6	1.489 (3)	Cl1—O5	1.4284 (16)
C5—H5A	0.9700	Cl1—O5^i^	1.4284 (16)
C5—H5B	0.9700	Cl1—O6	1.436 (2)
C6—O3	1.423 (2)	Cl1—O7	1.437 (2)
C6—H6A	0.9700	N1—H1A	0.8896
C6—H6B	0.9700	N1—H1B	0.8899
C7—O3	1.428 (2)	N1—H1C	0.8901
C7—C8	1.487 (3)	O1—C10^i^	1.427 (2)
C7—H7A	0.9700	O4—C5^i^	1.418 (2)
C7—H7B	0.9700		
			
C2—C1—C2^i^	121.7 (3)	C7—C8—H8A	109.9
C2—C1—Cl2	119.15 (13)	O2—C8—H8B	109.9
C2^i^—C1—Cl2	119.15 (13)	C7—C8—H8B	109.9
C1—C2—C3	119.1 (2)	H8A—C8—H8B	108.3
C1—C2—H2	120.5	O2—C9—C10	108.79 (17)
C3—C2—H2	120.5	O2—C9—H9A	109.9
C4—C3—C2	119.59 (19)	C10—C9—H9A	109.9
C4—C3—H3	120.2	O2—C9—H9B	109.9
C2—C3—H3	120.2	C10—C9—H9B	109.9
C3^i^—C4—C3	120.9 (2)	H9A—C9—H9B	108.3
C3^i^—C4—N1	119.52 (12)	O1—C10—C9	109.65 (16)
C3—C4—N1	119.52 (12)	O1—C10—H10A	109.7
O4—C5—C6	108.56 (15)	C9—C10—H10A	109.7
O4—C5—H5A	110.0	O1—C10—H10B	109.7
C6—C5—H5A	110.0	C9—C10—H10B	109.7
O4—C5—H5B	110.0	H10A—C10—H10B	108.2
C6—C5—H5B	110.0	O5—Cl1—O5^i^	110.74 (17)
H5A—C5—H5B	108.4	O5—Cl1—O6	109.74 (9)
O3—C6—C5	109.35 (15)	O5^i^—Cl1—O6	109.74 (9)
O3—C6—H6A	109.8	O5—Cl1—O7	109.15 (10)
C5—C6—H6A	109.8	O5^i^—Cl1—O7	109.15 (10)
O3—C6—H6B	109.8	O6—Cl1—O7	108.27 (14)
C5—C6—H6B	109.8	C4—N1—H1A	109.4
H6A—C6—H6B	108.3	C4—N1—H1B	109.5
O3—C7—C8	109.26 (15)	H1A—N1—H1B	109.5
O3—C7—H7A	109.8	C4—N1—H1C	109.5
C8—C7—H7A	109.8	H1A—N1—H1C	109.5
O3—C7—H7B	109.8	H1B—N1—H1C	109.5
C8—C7—H7B	109.8	C10—O1—C10^i^	112.8 (2)
H7A—C7—H7B	108.3	C9—O2—C8	113.21 (15)
O2—C8—C7	108.89 (15)	C6—O3—C7	111.95 (14)
O2—C8—H8A	109.9	C5—O4—C5^i^	112.76 (19)
			
C2^i^—C1—C2—C3	−0.6 (4)	O2—C9—C10—O1	−65.6 (2)
Cl2—C1—C2—C3	179.84 (17)	C9—C10—O1—C10^i^	175.35 (13)
C1—C2—C3—C4	0.4 (3)	C10—C9—O2—C8	−168.13 (16)
C2—C3—C4—C3^i^	−0.3 (4)	C7—C8—O2—C9	−179.95 (16)
C2—C3—C4—N1	−179.03 (18)	C5—C6—O3—C7	171.17 (16)
O4—C5—C6—O3	−66.0 (2)	C8—C7—O3—C6	177.55 (16)
O3—C7—C8—O2	65.9 (2)	C6—C5—O4—C5^i^	−172.01 (12)

Symmetry codes: (i) *x*, −*y*+1/2, *z*.

### Hydrogen-bond geometry (Å, °)

**Table:**

*D*—H···*A*	*D*—H	H···*A*	*D*···*A*	*D*—H···*A*
N1—H1A···O4	0.89	2.14	2.896 (3)	142.
N1—H1A···O3^i^	0.89	2.21	2.9311 (17)	138.
N1—H1B···O2^i^	0.89	2.14	2.8952 (18)	143.
N1—H1B···O1	0.89	2.18	2.870 (3)	134.
N1—H1C···O2	0.89	2.15	2.8952 (18)	140.
N1—H1C···O3	0.89	2.20	2.9311 (17)	139.

Symmetry codes: (i) *x*, −*y*+1/2, *z*.
